# Supplementary material for: Patterned crystal growth and heat wave generation in hydrogels
Source: Nat Commun. 2022 Jan 11;13:259. doi: 10.1038/s41467-021-27505-z (PMC8752664; doi:10.1038/s41467-021-27505-z)
Supplement: Supplementary file 1 — Supplementary Information [file 41467_2021_27505_MOESM1_ESM.pdf]

Supplementary Information for

Patterned crystal growth and heat wave generation in hydrogels

Thomas B. H. Schroeder[1]*, Joanna Aizenberg[1,2]*
1. John A. Paulson School of Engineering and Applied Science, Harvard University, Cambridge, MA
2. Department of Chemistry and Chemical Biology, Harvard University, Cambridge, MA
* corresponding authors – tschroeder@g.harvard.edu, jaiz@seas.harvard.edu

Contents

Section 1. Precursor solution compositions by weight and molarity 2
Supplementary Table 1. Precursor solution compositions. 2

Section 2. Electron microscopy and crystal size characterization 2
Supplementary Figure 1. SEM image of masked/unmasked interface and detail of unmasked region. 2
Supplementary Figure 2. SEM images of unmasked samples with varying acrylamide concentration. 3
Supplementary Table 2. Approximate domain/pore sizes shown in Supplementary Figures 2 and 3. 4
Supplementary Figure 3. Crystal/pore size ranges for various acrylamide concentrations. 4

Section 3. Consideration of mechanisms for polymerization-induced crystal growth inhibition....4
Supplementary Figure 3. Fits of various models to data from Figure 2A. 4
Supplementary Table 3. Fitting parameters for additive adsorption model. 6
Supplementary Table 4. Fitting parameters for pore penetration model. 7
Supplementary Figure 5. Simulations of growth arrest and stochastic nucleation mechanism. 9

Section 4. Derivation and fitting of temperature profiles 10
Supplementary Figure 6. Spatial temperature profiles of samples suspended in air. 11
Supplementary Figure 7. Formulation of heat transfer problem. 11
Supplementary Table 5: Definition and calculation of heat transfer parameters. 12
Supplementary Table 6. Fitting parameter values from global fits. 13
Supplementary Figure 8. Maximum temperature as a function of crystal growth front velocity. 13
Supplementary Figure 9. The effects of varying α, β, and γ. 14

Section 5. Other salts 14
Supplementary Table 7. Growth rate of other salt hydrate crystals in unpolymerized and polymerized acrylamide solutions. 14

Section 6. Consideration of spontaneous primary homogeneous nucleation 14

Section 7. References...15

Section 1. Precursor solution compositions by weight and molarity

When all components are fully dissolved, each solution has a final volume of 10 mL.

Supplementary Table 1. Precursor solution compositions.

**Sample composition (molar)**	**deionized water (mL)**	**anhydrous sodium acetate (g)**	**Acrylamide (g)**	***N,N'*-methylene-bisacrylamide (mg)**
7.0 M NaAc	6.3	5.75	--	--
7.0 M NaAc 0.7 M acrylamide	5.9	5.75	0.50	--
7.0 M NaAc 1.4 M acrylamide	5.4	5.75	1.0	--
7.0 M NaAc 2.1 M acrylamide	4.9	5.75	1.5	--
7.0 M NaAc 2.8 M acrylamide	4.4	5.75	2.0	--
7.0 M NaAc 2.8 M acrylamide 13 mM bis	4.4	5.75	2.0	20

Section 2. Electron microscopy and crystal size characterization

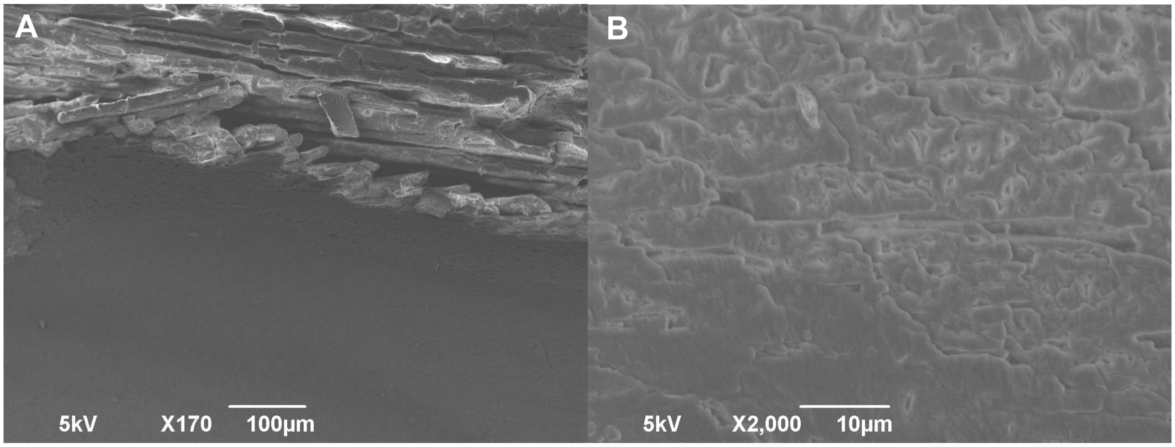

Supplementary Figure 1. Scanning electron micrographs of cured, crystallized sample containing 2.8 M acrylamide, 19.5 mM *N,N'*-methylenebisacrylamide, 7.0 M sodium acetate, and 2 mM α-ketoglutaric acid. **A**. Boundary between masked (top) and unmasked (bottom) areas. **B**. Detail of unmasked area.

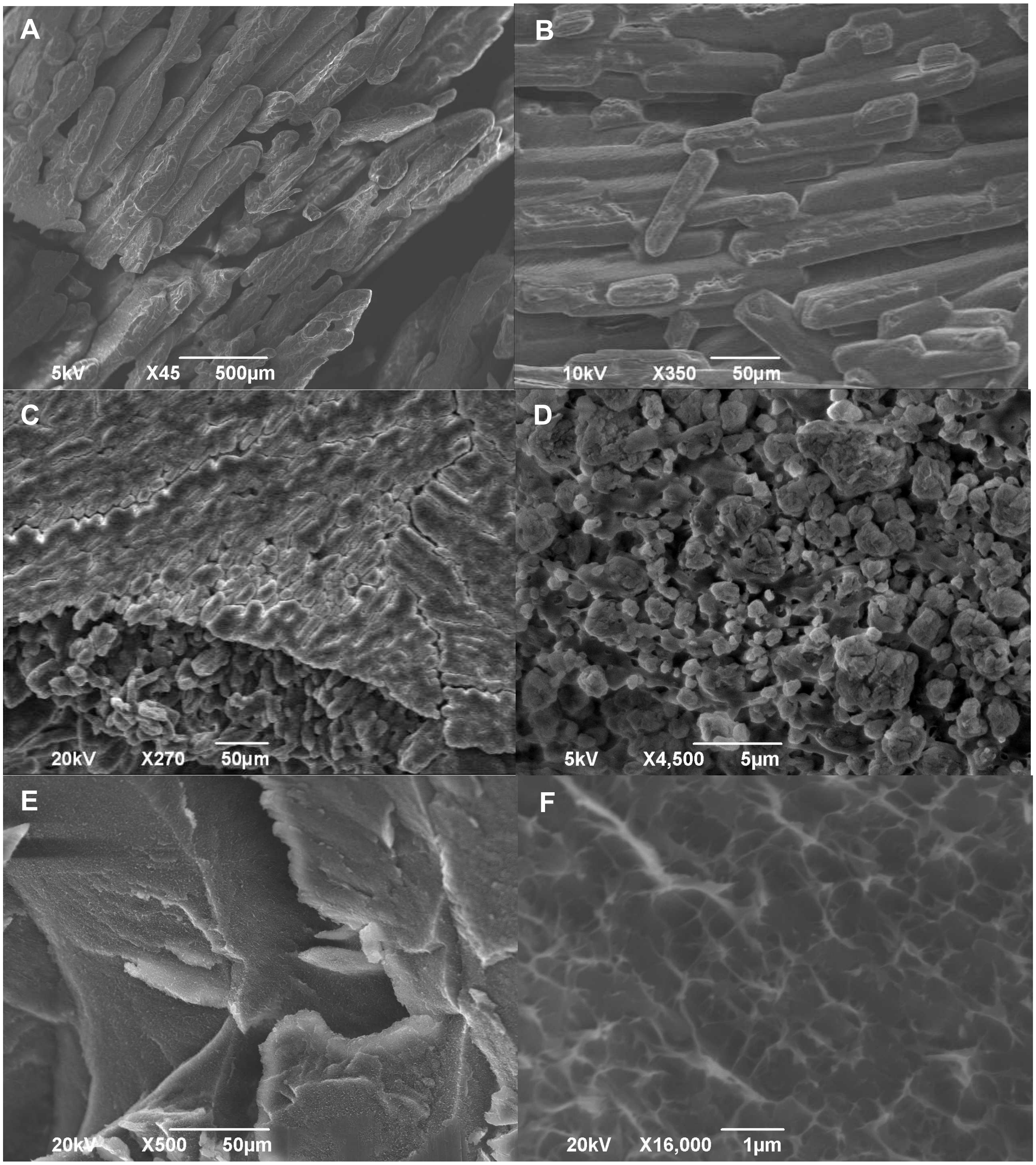

Supplementary Figure 2. Scanning electron micrographs of cured, crystalline samples containing 7.0 M sodium acetate and varying concentrations of acrylamide polymerized with 2 mM α-ketoglutaric acid and without a cross-linker. Approximate domain sizes are aggregated in **Supplementary Table 2**. **A**. No acrylamide. **B**. 0.7 M acrylamide. **C**. 1.4 M acrylamide (sectioned; corner visible). **D**. 2.1 M acrylamide (cross-section). **E**. 2.8 M acrylamide (cross-section; features clearly visible at this magnification are artifacts from sectioning). **F**. 2.8 M acrylamide (cross-section) at higher magnification.

Supplementary Table 2. Approximate diameters of pores or crystalline domains in polymer solutions with a range of acrylamide concentrations. Extracted from SEM images such as those in **Supplementary Figure 2**, plotted in **Supplementary Figure 3**.

**Acrylamide concentration (M)**	**Crystal domain or pore diameter (μm)**
0	80 - 200
0.7	20 - 120
1.4	10 - 40
2.1	0.5 - 5
2.8	0.1 - 1

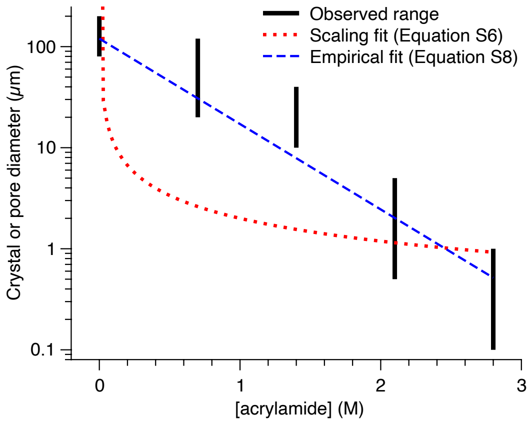

Supplementary Figure 3. Crystal/pore diameter ranges from **Supplementary Table 2** fit with Equations 6 (red dotted line) and 8 (blue dashed line). Crystal diameters were used for all acrylamide concentrations except 2.8 M, in which case crystal grains were indistinct and pore sizes were used.

Section 3. Consideration of mechanisms for polymerization-induced crystal growth inhibition

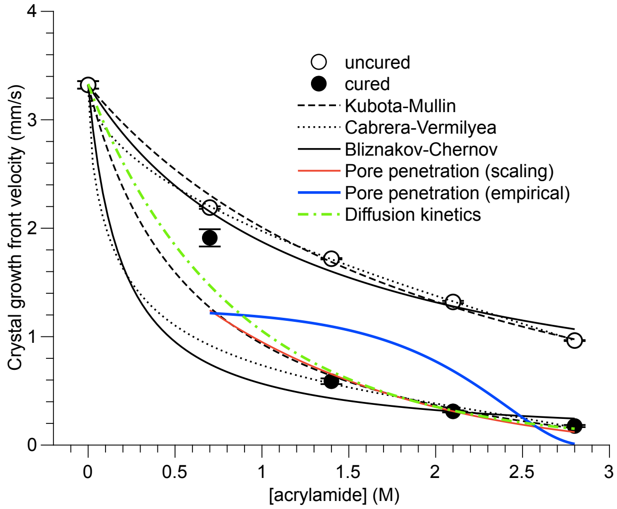

Supplementary Figure 4. Fits of various models described in this section to the data shown in **Figure 2A**. Error bars indicate standard error of the mean.

A. Additive adsorption mechanisms

One possible mechanism by which additives might inhibit crystal growth more strongly when polymerized than as monomers in solution involves binding to the surface of the growing crystal, blocking its progression. We have fit our sodium acetate trihydrate crystal growth front velocity data in the presence of unpolymerized acrylamide to three leading models of crystal growth kinetics in the presence of adsorbing additives (**Supplementary Figure 4**). These models differ slightly in their assumptions and functional forms as described below; an excellent overview of these models is provided in a monograph by Sangwal.[1] Each model expresses the average linear step growth velocity in the presence of additives (v) normalized by the velocity in the absence of impurities (v_0) in terms of the additive concentration (c_{add}), the affinity of the additive for the surface (K), and an effectiveness factor (α). The normalized average step velocity v/v_0 is assumed to equal the normalized average face growth velocity via the Burton-Cabrera-Frank screw dislocation mechanism.[2]

First, the model developed by Cabrera and Vermilyea[3] (Equation 1) assumes that additive particles adsorb to the surface terrace and are immobile compared to the velocity of the step ledges. These particles prevent the progression of step growth when they are spaced close enough together to prevent a two-dimensional crystal nucleus of critical size from squeezing between them. A geometric mean of the step velocity is used. The effectiveness factor $\alpha = \frac{r_{2D}^*}{\lambda} = \frac{a\gamma_l}{k_B T \lambda \sigma}$, where r_{2D}^* is the critical radius of a two-dimensional nucleus, λ is the average distance between adsorption sites on the surface, a is the diameter of a crystallizing molecule, γ_l is the linear edge free energy (related to the surface free energy γ by $\gamma_l = \gamma a^2$), k_B is the Boltzmann constant, T is the temperature, and σ is the relative supersaturation of the crystallizing species. In this model, $0 < \alpha < \infty$; additives with $\alpha > 0.5$ can arrest crystal growth entirely at sufficient concentration. Using a Langmuir adsorption isotherm, the normalized velocity is expressed as Equation 1.

$$\frac{v}{v_0} = \sqrt{1 - 2\alpha\sqrt{\frac{Kc_{add}}{1+Kc_{add}}}} \tag{1}$$

Next, a similar model developed by Kubota and Mullin[4] instead uses an arithmetic mean of the step velocity; further analysis by Sangwal asserts that this model also applies in the case of mobile additives, which would adsorb preferentially to kinks in step ledges and block their further progression.[1] The effectiveness factor takes the same form as the Cabrera-Vermilyea model: $\alpha = \frac{r_{2D}^*}{x_0} = \frac{a\gamma_l}{k_B T x_0 \sigma}$, where x_0 is the average distance between adsorption sites in the ledge (*i.e.* kinks). Again, $0 < \alpha < \infty$; however, in this model, fully arresting growth requires that $\alpha > 1$.

$$\frac{v}{v_0} = 1 - \alpha\frac{Kc_{add}}{1+Kc_{add}} \tag{2}$$

The earlier model developed by Bliznakov[5,6] and refined by Chernov[7] is also expressed using Equation 2, except that $0 < \alpha < 1$, implying that crystal growth impeded by this mechanism can

never be fully arrested. This formulation eschews a consideration of critical nuclei; instead, $\alpha = \frac{v_0 - v_i}{v_0}$, where v_i is the step growth rate in the presence of impurities at full coverage.

Each of these models fits well to our data from uncured solutions of acrylamide (**Supplementary Figure 4**, **Supplementary Table 3**). The models fit less well to the polymerized data sets; however, the free radical polymerization process used in this work produces a statistical distribution of molecular weights rather than monodisperse polymers. It may therefore be inappropriate to use the initial monomer concentration as c_{add} in these models. However, polymerization can be reasonably expected to increase the binding affinity of an additive through cooperative effects,[8,9] which is reflected in the fit parameters in **Supplementary Table 3**.

Supplementary Table 3. Fitting parameters for additive adsorption models.

	**Unpolymerized samples**		**Polymerized samples**	
**Model**	α	K **(M^{-1})**	α	K **(M^{-1})**
Cabrera-Vermilyea	0.688	0.283	0.514	5.68
Kubota-Mullin	1.255	0.460	1.17	1.61
Bliznakov-Chernov	0.982	0.796	0.991	5.14

B. Pore penetration mechanism

Another plausible mechanism by which polymeric additives may slow crystal growth requires considering the thermodynamics of crystallization in pores. When crystals are sufficiently small and have a high enough interfacial curvature (κ), the interfacial energy between the crystal and liquid phases, γ, contributes non-negligibly to the pressure inside the crystal p_c according to $p_c = p_L + \gamma\kappa$, where p_L is the pressure in the liquid. The normal melting point of a crystal, T_m, applies to infinitely large crystals with no curvature in contact with a liquid phase at its vapor pressure, p_e. The actual melting point $\tilde{T}_m$ of a crystal with finite curvature in a liquid whose pressure may deviate from p_e is expressed by Equation 3; this curvature-related difference is known as the Gibbs-Thomson effect and is reviewed along with other thermodynamic considerations of crystallization in porous media by Scherer[10,11] and more recently by Meldrum and O'Shaughnessy.[12] In this formula, v_L and v_c are the partial molar volumes of the liquid and crystalline phases and $\Delta S_{fus,v}$ is the entropy of fusion of the crystal per unit volume.

$$\tilde{T}_m - T_m = \left(\frac{v_L - v_c}{v_c}\right)\frac{p_L - p_e}{\Delta S_{fus,v}} - \frac{\gamma\kappa}{\Delta S_{fus,v}} \tag{3}$$

To penetrate a pore of diameter ξ, a crystal must adopt a curvature $\kappa = \frac{2}{\xi}$; if $p_L = p_e$, then

$$\tilde{T}_m = T_m - \frac{2\gamma}{\xi \Delta S_{fus,v}} \tag{4}$$

This reduction in the melting point reduces the degree of supercooling ($\Delta T = \tilde{T}_m - T$) at room temperature, which is the driving force for crystallization in supercooled melts. This driving force is generally related to the crystal growth rate by a function of the form $v = k(\Delta T)^g$, where k is a growth rate constant and g is the "order" of the crystallization process (distinct from "order" in

chemical kinetics; here g has no obvious fundamental meaning) where $1 \leq g \leq 2.5$.[13] For constant T, constructing a growth rate function in this way results in a function of the form:

$$v = k((T_m - T) - \frac{2\gamma}{\xi \Delta S_{fus,v}})^g \tag{5}$$

The reduction of the driving force in porous media owing to the Gibbs-Thomson effect therefore lowers the crystal growth front velocity as the pore size is reduced.

Even in the absence of cross-linker, sufficiently concentrated "semi-dilute" polymer solutions have a characteristic mesh size. ("Semi-dilute," in the parlance of polymer physics, indicates that the polymer fraction exceeds a threshold required for coils to overlap, but is much less than 1). The mesh size ξ of semi-dilute linear polymer coils in good solvent generally scales with the polymer fraction Φ of the polymer according to $\xi \cong a\Phi^{-3/4}$, where a is a constant, regardless of the degree of polymerization of the system.[14] In the solutions analyzed here, the molar concentration of acrylamide c is an approximately linear function of its mass fraction within the system (**Supplementary Information Section 1**), leading to Equation 6, which yields $v(c)$ expressed by Equation 7:

$$\xi = ac^{-3/4} \tag{6}$$

$$v = k((T_m - T) - Ac^{3/4})^g \tag{7}$$

where $A = \frac{2\gamma}{a\Delta S_{fus,v}}$.

Fitting Equation 7 to the rate data where polymers were present while setting $T_m - T$ to 38 K and allowing k, A, and g to vary yields the red curve shown in **Supplementary Figure 4** and the parameters shown in **Supplementary Table 4**. The resulting curve is very similar to the results of fitting the Kubota-Mullin model to the polymerized data.

Supplementary Table 4. Fitting parameters for pore penetration model using different methods of determining mesh size.

**Mesh size model**	***k***	***A***	***g***
Scaling (Eq. 6)	2.96×10^{-4}	12.4	2.49
Empirical (Eq. 8)	1.58×10^{-4}	0.14	2.47

However, the trend in crystal diameters observed via scanning electron microscopy (**Supplementary Figures 2 and 3, Supplementary Table 2**) as a function of acrylamide concentration is poorly fit by Equation 6 (**Supplementary Figure 3,** red dotted line), indicating that the crystals are not growing to perfectly occupy the pores presented by the polymer network and stopping upon reaching the pore diameter. The crystal diameter distribution is better described by the empirical relation in Equation 8 (**Supplementary Figure 3**, blue dashed line):

$$\xi \cong a * 10^{-bc} \tag{8}$$

where a = 120 μm and b = 0.845.

Substituting Equation 8 into Equation 5 yields Equation 9:

$$v = k((T_m - T) - A * 10^{bc})^g \quad (9)$$

where $A = \frac{2\gamma}{a\Delta S_{fus,v}}$. Fitting Equation 9 to the rate data where polymers were present while setting $T_m - T$ to 38 K, b to 0.845 (derived from the empirical fit above) and allowing k, A, and g to vary yields the blue curve shown in **Supplementary Figure 4** and the parameters shown in **Supplementary Table 4**. The quality of this fit is noticeably worse than that of Equation 7; other functions that empirically fit the size data as a function of acrylamide concentration in **Supplementary Figure 3** and **Supplementary Table 2** well perform similarly poorly when substituted into Equation 5 and fit to the rate data. These poor fits may point away from the Gibbs-Thomson effect as a significant driver of the trend observed here.

Note that in this section we have considered salt hydrates as supercooled melts. Similar formulations exist for crystal growth from supersaturated solutions (e.g. $v = k(\Delta c)^g$), where the driving gradient is the degree of supersaturation. Salt hydrates such as sodium acetate trihydrate, in which solvating water co-crystallizes with the solute to form solid crystals that melt at 58°C at standard pressure, may be fairly considered in either way; in this case, it is simpler to consider them as melts.

C. Growth arrest and stochastic nucleation mechanism

The descriptions above present means by which the growth rates of single crystals may be suppressed in the presence of polymers. However, **Figure 1C** and **Supplementary Figures 2** and **3** make clear that polymerized samples form highly polycrystalline composites with a crystallite size range that decreases with increasing polymer concentration. A mechanism that takes this phenomenon into account may explain the observed growth rate trends.

Invoking an argument first presented by Chernov,[7] Asenath-Smith *et al.*[15] have proposed that crystals growing into polymer fibers may experience an abrupt increase in resistance to mass transport to the interface. As sodium acetate crystals grow in a needle shape, the active growth face has a small area; small crystallites in particular may be fully occluded upon contact with the polymer mesh and halt their lengthwise growth completely. In such a case, the growth front would proceed only due to the continuous stochastic secondary nucleation of new crystallites.

We have simulated this scenario using MATLAB (code available with manuscript). To initialize the simulation, a predetermined polymer volume fraction is distributed across random voxels throughout a sample volume and initial crystal "seeds" are planted at one end. A loop of timesteps follows; during each timestep, a) each active crystal grows by a length unit, b) each crystal that has collided with a polymer, another crystal, or the sample volume boundary is rendered inactive, and c) each voxel occupied by a crystal has a set probability of nucleating a new crystal with a random orientation. A snapshot of a simulated volume containing polymers (red) and crystals (blue) is shown in **Supplementary Figure 5A**. At each timestep, the position is recorded of the farthest plane from the starting point in which a threshold percentage of the voxels have been

converted to crystal; thresholds of 10%, 1%, 0.1%, 0.01% are shown in black. By inspection, after a sufficient induction time has passed, the average rate of progression of these threshold planes are constant and equal to one another regardless of the threshold percentage (**Supplementary Figure 5B**). Induction times increase with increased threshold percentage; the velocities of higher threshold planes over time fluctuate less than in lower thresholds. In order to analyze front velocity trends, we tracked the velocity of the 1% threshold plane during the final half of growth simulations which ran for more than twice this induction time. We used a 100 x 100 x (variable length) sample volume; 25 initial seeds were evenly placed along the initial plane during initialization and randomly oriented at angles of more than 45° from the direction of front progression. We ran 3 simulations for each set of parameters.

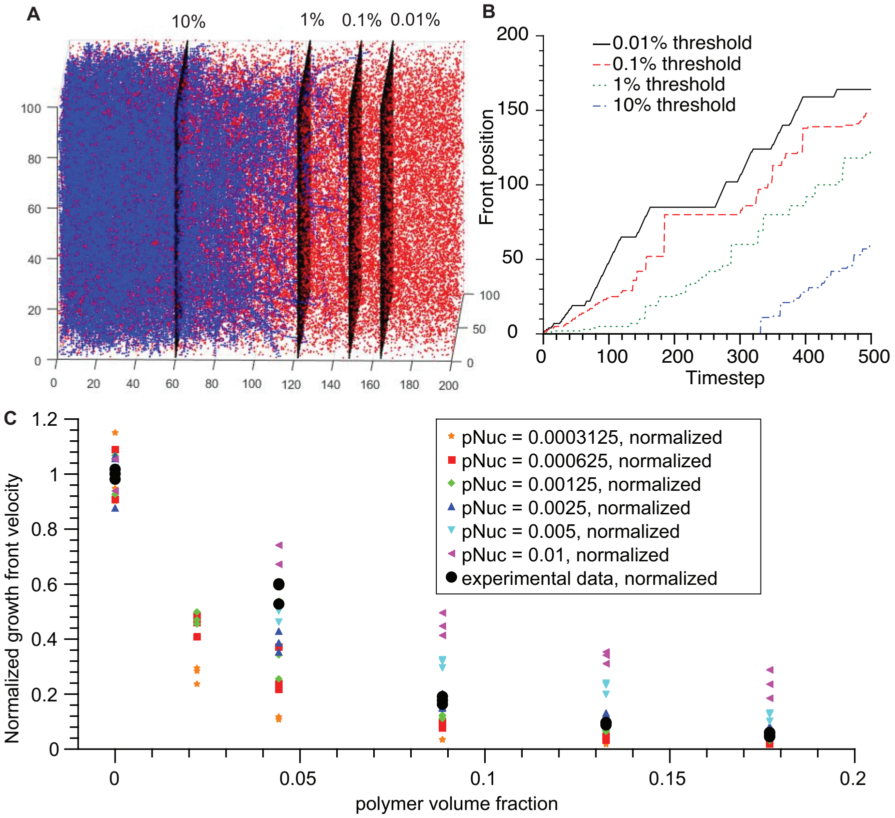

Supplementary Figure 5. Simulations of growth arrest and stochastic nucleation mechanism. **A**. Snapshot of simulation with a polymer volume fraction of 0.022125 and a nucleation probability per timestep of 0.00125. Red voxels are polymer, blue voxels are crystal, and black voxels indicate various threshold planes. **B**. Progress of threshold planes over time in the simulation shown in A. **C**. Simulated crystal growth front velocities at different polymer volume fraction and nucleation probability per timestep values overlaid with experimental rate data from **Figure 2A**, assuming 100% polymerization. $N = 3$ for all values.

The two variables that affect the growth rate in simulated systems containing polymers relative to simulated systems without polymers are the polymer volume fraction and the probability of nucleation per timestep. The effects of these parameters on front velocity are shown in **Supplementary Figure 5C**. Growth front rates are depressed at increased polymer volume fraction; this dependence is reduced as nucleation rates are raised. **Supplementary Figure 5C** is

overlaid with experimental data from **Figure 2A** (assuming 100% polymerization and that the density of polyacrylamide in solution is the same as that of solid acrylamide monomers). While no one parameter set fits the experimental data perfectly, the overall trend of a monotonically decreasing front velocity with increasing polymer concentration is captured.

D. Viscosity/diffusion kinetics mechanism

Finally, polymerizing the acrylamide in a solution drastically increases its viscosity (captured in the denominator of the parameter k in Equation 5). The various mass transfer processes involved in crystal growth (*e.g.* diffusion and orientation of solute ions) will likely be slower in polymerized solutions, slowing the velocity of the growth front. Polymer solutions are complex fluids in which viscous interactions are dependent on both length and time scales;[16,17] untangling this contribution to crystal growth inhibition is non-straightforward. Yang *et al*. have proposed applying a stretched exponential model to a similar system in which the rate is proportional to the diffusion coefficient *D*, which relates to the polymer fraction Φ according to $D \propto e^{-a\Phi^{b}}$ where a and b are empirical fitting parameters.[18] Assuming again that Φ is proportional to the molar acrylamide concentration c yields Equation 10:

$$v = v_0 e^{-ac^{b}} \tag{10}$$

When fit to the cured solutions' growth rate data, this equation produced the green dash-dot line in **Supplementary Figure 4**, which agrees with the data rather well and yields the following fitting parameters: *a* = 1.15; *b* = 0.96.

Section 4. Derivation and fitting of temperature profiles

The following derivation takes as its starting point the formulation that Hopper and Uhlmann (1973) solved for the one-dimensional temperature distribution around the moving crystal-solution interface during crystal growth at a constant velocity.[19] These authors found a steady state solution for the temperature profile within the liquid phase, but none exists for the temperature profile in the solid phase under the conditions they describe, which includes no heat transfer outside the system. This result is consistent with our observations of crystallizing systems suspended in air (**Supplementary Figure 6**), in which temperatures at and behind the moving front rise over time. However, we observed that crystallizing systems on a cold plate held to a constant temperature quickly reach a temperature profile around the solid-liquid interface that remains stable over time (**Figure 3A,B**). When formulating the heat transfer problem under these conditions, we include a dissipation term representing conductive heat transfer to the cold plate (normal to the direction of crystal growth) that was absent in Hopper and Uhlmann's analysis; this addition gives rise to a steady state solution (Equations 18 and 19).

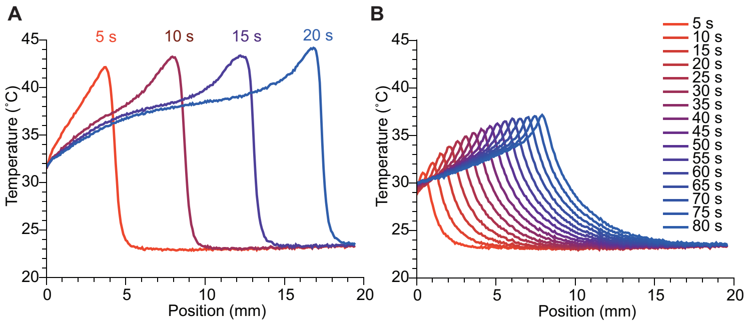

Supplementary Figure 6. Spatial temperature profiles at 5-second intervals resulting from growth of sodium acetate trihydrate crystals through aqueous solutions of unpolymerized (**A**) and polymerized (**B**) additives in samples suspended in the air. Precursor solution composition for all samples: 7.0 M sodium acetate, 2.8 M acrylamide, 13 mM *N,N'*-methylenebisacrylamide, 2 mM α-ketoglutaric acid in water. Profiles do not reach a steady state, contrasting with **Figure 3A,B**.

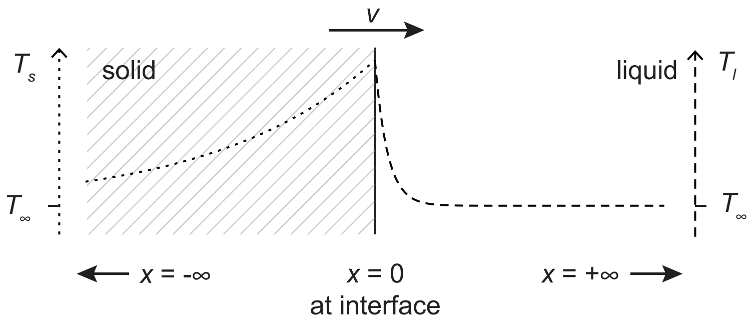

Supplementary Figure 7. Formulation of heat transfer problem.

In this analysis, we derive the one-dimensional temperature distributions in the solid and liquid phase ($T_s(x)$, $T_l(x)$) in a crystallizing supercooled system on a cold plate held at temperature T_∞ with a frame of reference centered around a solid-liquid boundary ($x = 0$) moving with constant velocity v (**Supplementary Figure 7**). The system is solid in the negative domain ($-\infty < x \leq 0$) and liquid in the positive domain ($0 \leq x < \infty$). Equations 11 and 12 quantify heat conduction,[20] Equations 13-15 are boundary conditions, and Equation 16 is a heat balance at the interface. In the equations below, α (m^2 s^{-1}) is the thermal diffusivity, β (K) is the latent heat scaled by specific heat capacity, and γ (s^{-1}) is the thermal contact conductance of the interface between the system and the cold plate scaled by heat capacity (see **Supplementary Table 5** for formal definitions). For simplicity, these parameters are assumed to be identical between the solid and liquid states.

Supplementary Table 5: Definition and calculation of heat transfer parameters using literature values. Below, k is thermal conductivity, ρ is density, C_p is specific heat capacity at constant pressure, ΔH_{fus} is enthalpy of fusion, h is heat sink contact conductance, z is sample thickness.

Parameter	Formula	Literature values
α	$\alpha = \frac{k}{\rho C_p}$	k = 0.67 W m^{-1} K^{-1} (45.3 mass% sodium acetate, solid, at 300 K)[21]<br>ρ = 1372 kg m^{-3} (pure)[22]<br>C_p = 2.2 kJ kg^{-1} K^{-1} (54.3 mass% sodium acetate, solid, at 303 K)[21]<br>→ α_{lit} = 2.2 x 10^{-7} m^2 s^{-1}
β	$\beta = \frac{\Delta H_{fus}}{C_p}$	C_p as above; ΔH_{fus} = 100 kJ kg^{-1} (47.5 mass%, 303 K)[21]<br>→ β_{lit} = 45 K
γ	$\gamma = \frac{h}{z\rho C_p}$	n/a. h has units of J m^{-2} s^{-1} K^{-1}; z has units of m.

$$\alpha \frac{d^2T_s}{dx^2} + \gamma(T_s - T_0) = v\frac{dT_s}{dx} \tag{11}$$

$$\alpha \frac{d^2T_l}{dx^2} + \gamma(T_l - T_0) = v\frac{dT_l}{dx} \tag{12}$$

$$T_s(-\infty) = T_\infty \tag{13}$$

$$T_l(\infty) = T_\infty \tag{14}$$

$$T_s(0) = T_l(0) \tag{15}$$

$$\left.\frac{dT_s}{dx}\right|_{x=0} - \left.\frac{dT_l}{dx}\right|_{x=0} = \frac{\beta}{\alpha}v \tag{16}$$

Equations 11 and 12 each have solutions of the form shown in Equation 17 where C_1 and C_2 are constants:

$$T(x) = T_\infty + C_1 e^{-\frac{v}{2\alpha}\left(1+\sqrt{1+\frac{4\alpha\gamma}{v^2}}\right)x} + C_2 e^{-\frac{v}{2\alpha}\left(1-\sqrt{1+\frac{4\alpha\gamma}{v^2}}\right)x} \tag{17}$$

After applying boundary conditions 13-16, Equations 18 and 19 emerge as solutions:

$$T_s(x) = T_\infty + \frac{\beta}{\sqrt{1+\frac{4\alpha\gamma}{v^2}}} e^{\frac{-v}{2\alpha}\left(1-\sqrt{1+\frac{4\alpha\gamma}{v^2}}\right)x}, \; (-\infty < x \leq 0) \tag{18}$$

$$T_l(x) = T_\infty + \frac{\beta}{\sqrt{1+\frac{4\alpha\gamma}{v^2}}} e^{\frac{-v}{2\alpha}\left(1+\sqrt{1+\frac{4\alpha\gamma}{v^2}}\right)x}, \; (0 \leq x < \infty) \tag{19}$$

Globally fitting these equations to the profiles at the final timepoints shown in **Figure 3A**,**B** with v fixed to the recorded front velocities (In **Figure 3A**, $v = v_{unpoly} = 0.896$ mm s^{-1}; in **Figure 3B**, $v = v_{poly} = 0.131$ mm s^{-1}) and α, β, and γ allowed to vary (but shared between data sets) yielded the dashed curves in the figures and fitting parameter values listed in **Supplementary Table 6**, which displayed reasonable agreement with literature values (which were calculated based on parameters collected for sodium acetate trihydrate in somewhat different conditions, making some discrepancy unavoidable; see **Supplementary Table 5**). Profiles for a variety of velocities with α, β, and γ fixed to the fit values are shown in **Figure 3C**. The sharp peak present in the model fit is blunted in the experimental data for the unpolymerized sample; this may be explained by a rough (non-discrete in x) advancing front or the diffusion of this steep gradient within the thickness of the cover glass between the crystallizing solution and the infrared camera.

Supplementary Table 6: Fitting parameter values from global fits of curves from **Figure 3 A,B**. See **Supplementary Table 5** for calculation of reference values from literature values.

**Parameter**	**Value from fit**	**Reference value**
α	4.2 x 10^{-7} m^2 s^{-1}	2.2 x 10^{-7} m^2 s^{-1}
β	34 K	45 K
γ	0.175 s^{-1}	

It is clear that varying the crystal growth front velocity alone – which can be achieved by varying the composition and polymerization state of the additives to the solution – can be responsible for large changes in the temperature profile of the system. The maximum temperature can be expressed as a function of crystal growth front velocity using Equation 20. This is plotted in **Supplementary Figure 8**; it can be observed that $\lim_{v\to\infty} T_{max} = T_\infty + \beta$ = 55˚C. Based on prior reports of crystallization in metastable sodium acetate solutions diluted beyond a 1:3 salt:water stoichiometric mixture,[23,24] we expect this temperature to approach but never reach sodium acetate trihydrate's melting point of 58 ˚C, which agrees with this result. The effects of varying α, β, and γ are shown in **Supplementary Figure 9**.

$$T_{max} = T_s(0) = T_l(0) = T_\infty + \frac{\beta}{\sqrt{1+\frac{4\alpha\gamma}{v^2}}} \qquad (20)$$

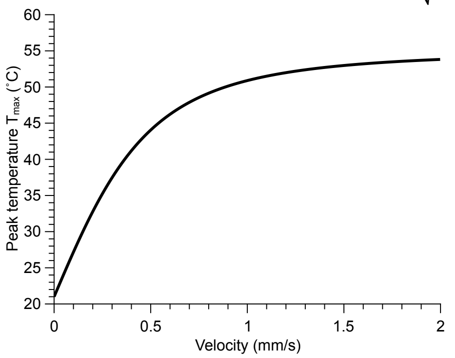

Supplementary Figure 8. Maximum temperature varies as a function of crystal growth front velocity.

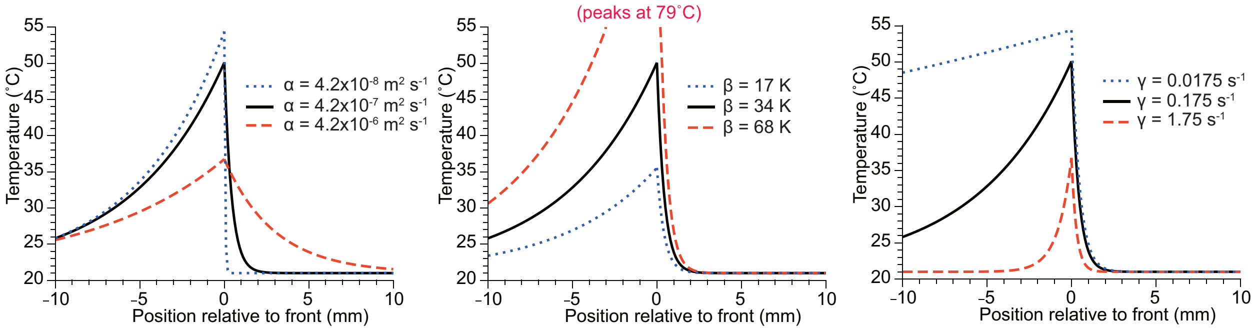

Supplementary Figure 9. The effects of varying α, β, and γ. In each plot, black solid lines show the temperature profile computed from Equations 18 and 19 using fitting parameters from the uncured sample (compiled in **Supplementary Table 6**), blue dotted lines show the temperature profile when the parameter of interest is reduced by some factor, and red dashed lines show the temperature profile when the parameter of interest is increased by the same factor (10 for α and γ, 2 for β).

Section 5. Other salts

Supplementary Table 7: Growth rate of other salt hydrate crystals in unpolymerized and polymerized acrylamide solutions. Polymerization was achieved via UV irradiation for 10 minutes using 20 mM α-ketoglutaric acid. Average rates ± s.e.m. shown (where applicable); N = 2 or 3.

Salt hydrate	Melting point (˚C)	[salt] (M)	[acryl-amide] (M)	Crystal growth rate through unpolymerized solution (μm/s)	Crystal growth rate through polymerized gel (μm/s)	Notes
$Mg(NO_3)_2{\bullet}6H_2O$	89	3.7	2.6	91 ± 0.3	2.6 ± 0.1	Nucleation in polymerized case proceeded spontaneously from locations in channel interior following cooling to 20˚C on Peltier plate.
$Na_2S_2O_3{\bullet}5H_2O$	48	3.5	1.9	120	5.9 ± 2.2	Gelation was noticeably inhibited.
$Ca(NO_3)_2{\bullet}4H_2O$	43	5.7	3.0	59 ± 3	No visible growth after 6 hours	

Section 6. Consideration of spontaneous primary homogeneous nucleation

According to classical nucleation theory, so-called "primary homogeneous nucleation" from the spontaneous assembly of a cluster of the crystallizing solid that exceeds the "critical radius" (beyond which the continued growth of the crystal is thermodynamically favorable) is a stochastic process; the probability of this occurring within a given time interval is determined by the degree of supersaturation or supercooling, the temperature, the molecular volume of the crystallizing species, the interfacial tension between solid and liquid states, the viscosity, and the volume of the solution.[13] Assuming the classical theory applies, one could therefore track a population of vials

that each contained a given volume of a solution with the same parameters, and the number of vials that had spontaneously crystallized over time should approximate the cumulative density function of a Poisson distribution. However, for the solutions and volumes described in this work, the timescales involved are long enough that spontaneous nucleation is observed infrequently; anecdotally, we have vials of uncrystallized metastable precursor solutions that have been lying around the lab at or below room temperature for over a year, an observation corroborated by Sandnes and Rekstad.[24] That said, it does happen -- unperturbed precursor solution vials are occasionally observed to crystallize on a timescale of days or weeks; accordingly, we recommend dividing precursor solutions into aliquots to hedge against this possibility. Deployments of metastable solutions in large contiguous quantities may be limited by primary homogeneous nucleation. However, commercially available hand warmers containing over 100 mL of supersaturated sodium acetate solution (2-3 orders of magnitude more than used in this work) resist nucleation well enough to be useful at winter temperatures while being carried outdoors, indicating that even quite substantial amounts of solution can be stable enough for applications where the cost of accidental crystallization is minimal.

Section 7. References

1. Sangwal, K. *Additives and Crystallization Processes*. (John Wiley & Sons, Ltd, 2007). doi:10.1002/9780470517833.
2. Burton, W. K., Cabrera, N., Frank, F. C. & Mott, N. F. The growth of crystals and the equilibrium structure of their surfaces. *Philos. Trans. R. Soc. Lond. Ser. Math. Phys. Sci.* **243**, 299–358 (1951).
3. Cabrera, N. & Vermilyea, D. A. The growth of crystals from solution. in *Growth and Perfection of Crystals: Proceedings of an International Conference on Crystal Growth, Cooperstown, New York, August 1958* (eds. Doremus, R. H., Roberts, B. W. & Turnbull, D.) 393–410 (Wiley, 1958).
4. Kubota, N. & Mullin, J. W. A kinetic model for crystal growth from aqueous solution in the presence of impurity. *J. Cryst. Growth* **152**, 203–208 (1995).
5. Bliznakov, G. M. Über die Wachstumsformen der Kristalle und den Einfluß der Adsorption auf die lineare Kristallisationsgeschwindigkeit. *Bull Acad Sci Bulg Ser Phy* **4**, 135–152 (1954).

6. Bliznakov, G. & Kirkova, E. Der Einfluß der Adsorption auf das Kristallwachstum. *Z. Für Phys. Chem.* **206O**, 271–280 (1956).

7. Chernov, A. A. *Modern Crystallography III: Crystal Growth*. (Springer, 1984).

8. Elhadj, S. *et al.* Peptide Controls on Calcite Mineralization: Polyaspartate Chain Length Affects Growth Kinetics and Acts as a Stereochemical Switch on Morphology. *Cryst. Growth Des.* **6**, 197–201 (2006).

9. Shtukenberg, A. G., Ward, M. D. & Kahr, B. Crystal Growth with Macromolecular Additives. *Chem. Rev.* **117**, 14042–14090 (2017).

10. Scherer, G. W. Freezing Gels. *J. Non-Cryst. Solids* **155**, 1–25 (1993).

11. Scherer, G. W. Crystallization in pores. *Cem. Concr. Res.* **29**, 1347–1358 (1999).

12. Meldrum, F. C. & O'Shaughnessy, C. Crystallization in Confinement. *Adv. Mater.* **32**, 2001068 (2020).

13. Mullin, J. W. *Crystallization*. (Butterworth-Heinemann, 2001).

14. de Gennes, P. G. *Scaling concepts in polymer physics*. (Cornell University Press, 1979).

15. Asenath-Smith, E., Li, H., Keene, E. C., Seh, Z. W. & Estroff, L. A. Crystal Growth of Calcium Carbonate in Hydrogels as a Model of Biomineralization. *Adv. Funct. Mater.* **22**, 2891–2914 (2012).

16. Makuch, K., Hołyst, R., Kalwarczyk, T., Garstecki, P. & F. Brady, J. Diffusion and flow in complex liquids. *Soft Matter* **16**, 114–124 (2020).

17. Amsden, B. Solute Diffusion within Hydrogels. Mechanisms and Models. *Macromolecules* **31**, 8382–8395 (1998).

18. Yang, F. (Kuo), Cholewinski, A., Yu, L., Rivers, G. & Zhao, B. A hybrid material that reversibly switches between two stable solid states. *Nat. Mater.* **18**, 874 (2019).

19. Hopper, R. W. & Uhlmann, D. R. Temperature distributions during crystallization at constant velocity. *J. Cryst. Growth* **19**, 177–186 (1973).

20. Deen, W. M. *Analysis of Transport Phenomena*. (Oxford University Press, 2012).

21. Araki, N., Futamura, M., Makino, A. & Shibata, H. Measurements of thermophysical properties of sodium acetate hydrate. *Int. J. Thermophys.* **16**, 1455–1466 (1995).

22. He, Y. *et al.* Improvement of supercooling and thermal conductivity of the sodium acetate trihydrate for thermal energy storage with α-Fe2O3 as addictive. *J. Therm. Anal. Calorim.* **133**, 859–867 (2018).

23. Desgrosseilliers, L., Allred, P., Groulx, D. & White, M. A. Determination of enthalpy–temperature–composition relations in incongruent-melting phase change materials. *Appl. Therm. Eng.* **61**, 193–197 (2013).

24. Sandnes, B. & Rekstad, J. Supercooling salt hydrates: Stored enthalpy as a function of temperature. *Sol. Energy* **80**, 616–625 (2006).
