# Supplementary material for: Patterned crystal growth and heat wave generation in hydrogels
Source: Nat Commun. 2022 Jan 11;13:259. doi: 10.1038/s41467-021-27505-z (PMC8752664; doi:10.1038/s41467-021-27505-z)
Supplement: Supplementary file 3 — Description of Additional Supplementary Files [file 41467_2021_27505_MOESM3_ESM.pdf]

Description of Additional Supplementary Files

File Name: Supplementary Movie 1

Description: **Crystallization of a metastable solution polymerized through a photomask.** Sample was exposed to UV light in all areas outside the letter H, which was covered by a mask. Precursor solution composition: 2.8 M acrylamide, 26 mM *N*,*N*'-methylenebisacrylamide, 7.0 M sodium acetate, 2 mM α-ketoglutaric acid. This video also demonstrates the procedure for crystallizing samples suspended in air; a paper grid supports the sample. Ruler shown for scale; numbered increments are centimeters. Video is shown at 10x speed.

File Name: Supplementary Movie 2

Description: **Video microscopy (10x) through crossed linear polarizers showing progression of crystal growth along interface between masked and unmasked regions.** Video shown at 10x magnification and 0.2x speed with automatic exposure. First frame shows faint phase boundary between masked (left) and unmasked (right) regions before growth front reaches field of view. Crystals grow rapidly through masked region with large, needle-like domains, then slowly outward through unmasked region where additives have been photopolymerized, forming small crystallites. Electron micrographs of similar samples are shown in Supplementary Figure 1. After some time has passed, a secondary cascade of small crystallites engulfs the large needles in the masked region; this corresponds to the growth of bright white areas visible in Figure 4A and Supplementary Movie 4. Secondary crystallite growth appears to be restricted to masked areas. Precursor solution composition: 2.8 M acrylamide, 26 mM *N*,*N*'-methylenebisacrylamide, 7.0 M sodium acetate, 2 mM α-ketoglutaric acid.

File Name: Supplementary Movie 3

Description: **Video microscopy (5x) through crossed linear polarizers showing progression of crystal growth along interface between masked and unmasked regions.** Video shown at 5x magnification and 0.2x speed with automatic exposure. See caption from Supplementary Movie 2. This video also shows converging fronts and bubble formation, which is common in regions where fronts converge and hypothesized to be a result of gel contraction during crystallization due to the higher density of the solid phase.

File Name: Supplementary Movie 4

Description: **Visible, infrared, and threshold temperature videos of crystal growth in hydrogel polymerized through "hello" photomask.** 10x speed; sample was crystallized on a Peltier cold plate held at 18˚C. Precursor solution composition: 2.8 M acrylamide, 13 mM *N*,*N*'-methylenebisacrylamide, 7.0 M sodium acetate, 2 mM α-ketoglutaric acid. Threshold temperature shown: 34.5 ˚C.

File Name: Supplementary Movie 5

Description: **Visible, infrared, and threshold temperature videos of crystal growth in hydrogel polymerized through hexagonal grid photomask.** 10x speed; sample was crystallized while suspended in air on a paper grid, slowing heat dissipation in areas with converging fronts. Precursor solution composition: 2.8 M acrylamide, 13 mM *N*,*N*'-methylenebisacrylamide, 7.0 M sodium acetate, 2 mM α-ketoglutaric acid. Threshold temperature shown: 42.5 ˚C.

File Name: Supplementary Movie 6

Description: **Confocal Z-stack showing crystallization-induced wave of thermoresponsive gel contraction.** 30x speed. Top-left is XZ plane (1.28 x 0.24 mm), bottom-left is XY plane (1.28 x 1.28 mm), bottom-right is YZ plane (0.24 x 1.28 mm). Film composition: *N*-isopropylacrylamide and Nile blue acrylamide cross-linked with polyethylene glycol diacrylate in water, frozen and thawed twice to induce microporosity and improve swelling kinetics. Fluorescence emission appears in red (λ_{ex} = 488 nm); transmitted light appears in white and vanishes once crystals pass under sample. Metastable hydrogel precursor solution composition: 2.8 M acrylamide, 19.5 mM *N*,*N*'-methylenebisacrylamide, 7.0 M sodium acetate, 2 mM α-ketoglutaric acid; 2 mm spacer. Channel below area on screen was unmasked during polymerization.

File Name: Supplementary Movie 7

Description: **Confocal Z-stack showing crystallization-induced wave of thermoresponsive gel contraction.** 35x speed. Top-left is XZ plane (2.56 x 0.26 mm), bottom-left is XY plane (2.56 x 2.56 mm), bottom-right is YZ plane (0.26 x 2.56 mm). Film composition: *N*-isopropylacrylamide and Nile blue acrylamide cross-linked with polyethylene glycol diacrylate in water, frozen and thawed twice to induce microporosity and improve swelling kinetics. Fluorescence emission appears in red (λ_{ex} = 488 nm). Metastable hydrogel precursor solution composition: 2.8 M acrylamide, 19.5 mM *N*,*N*'-methylenebisacrylamide, 7.0 M sodium acetate, 2 mM α-ketoglutaric acid; 2 mm spacer. Channel below area on screen was unmasked during polymerization.

File Name: Supplementary Movie 8

Description: **Confocal Z-stack showing selective gel contraction over masked region of crystallizing sample.** 13x speed. Top-left is XZ plane (2.56 x 0.30 mm), bottom-left is XY plane (2.56 x 2.56 mm), bottom-right is YZ plane (0.30 x 2.56 mm). Film composition: *N*-isopropylacrylamide and Nile blue acrylamide cross-linked with polyethylene glycol diacrylate in water, frozen and thawed twice to induce microporosity and improve swelling kinetics. Fluorescence emission appears in red (λ_{ex} = 488 nm). Metastable hydrogel precursor solution composition: 2.8 M acrylamide, 19.5 mM *N*,*N*'-methylenebisacrylamide, 7.0 M sodium acetate, 2 mM α-ketoglutaric acid; 0.5 mm spacer. Channel below area on screen was masked on left side during polymerization.

File Name: Supplementary Movie 9

Description: **Wax melting and wetting processes patterned using heat from crystal growth in hydrogel polymerized through "HOT" photomask.** 10x speed. A layer of wax had been deposited onto the underside of the paper, which was attached using thermally conductive glue to the top of a channel bearing a patterned metastable hydrogel. Sample was crystallized on a Peltier cold plate held at 20˚C. Wax composition: 1% (w/v) Oil Blue N in icosane. Hydrogel precursor solution composition: 2.8 M acrylamide, 19.5 mM *N*,*N*'-methylenebisacrylamide, 7.0 M sodium acetate, 2 mM α-ketoglutaric acid.

File Name: Supplementary Data 1

Description: Data shown in Figures 2 and 3A,B, Supplementary Figures 3, 5B,C, and 6A,B, and Supplementary Table 7.

File Name: Supplementary Software 1

Description: MATLAB code used in Supplementary Information Section 3C to simulate growth arrest and stochastic secondary nucleation.
